# Correction: Dreismickenbecker et al. Electroencephalography-Based Effects of Acute Alcohol Intake on the Pain Matrix. *Brain Sci.* 2023, *13*, 1659

**DOI:** 10.3390/brainsci14040363

**Published:** 2024-04-08

**Authors:** Elias Dreismickenbecker, Sebastian Zinn, Mara Romero-Richter, Madeline Kohlhaas, Lukas R. Fricker, Silvana Petzel-Witt, Carmen Walter, Matthias Kreuzer, Stefan W. Toennes, Malte Anders

**Affiliations:** 1 Center for Pediatric and Adolescent Medicine, Department of Pediatric Hematology/Oncology, University Medical Center Mainz, 55131 Mainz, Germany; 2 Clinical Development and Human Pain Models, Fraunhofer Institute for Translational Medicine and Pharmacology ITMP, 60596 Frankfurt, Germany; 3 Department of Anesthesiology, Intensive Care Medicine and Pain Therapy, Goethe University Frankfurt, University Hospital, Theodor-Stern-Kai 7, 60590 Frankfurt, Germany; 4 Institute of Legal Medicine, University Hospital, Goethe University, 60590 Frankfurt, Germany; 5 Department of Anesthesiology and Intensive Care, School of Medicine and Health, Technical University of Munich, 81675 Munich, Germany

## Missing Citation

This paper [1] was part of the methodology establishment within a study series. Therefore, the methodology of [36] and [1] is described in close alignment. However, in the original publication, [36] was not cited. 

The citation has now been inserted in Section 2.3. EEG Recording and Pre-Processing and should read: 

“We then epoched the data from −1 s to +2 s around the onset of each stimulus and subsequently calculated the event-related spectral perturbation (ERSP) and the inter-trial coherence (ITC) using EEGLAB’s newtimef-function with a divisive baseline from −1 s to 0 s, a resolution in time of 400 points from −1 s to +2 s, and a frequency resolution of 200 points between the frequencies of 3 Hz and 100 Hz [36–38]. We ran the wavelet transform portion of the newtimef-function with three cycles at the lowest frequency of 3 Hz and 20 cycles at the highest frequency of 100 Hz and analyzed the data at the electrode location Cz [31,36,39,40].”

The citation has also been inserted in Section 2.4. Statistics and should read:

“To account for multiple comparisons, instead of a common approach of an alpha level adjustment, we have applied a cluster-based approach as it has been used in the literature both for 2-dimensional [45,46] and 3-dimensional [47,48] EEG data. We have only reported results as being significant if they appeared in clusters of at least 3 × 3 pixels in size [36].”

With this correction, the order of some references has been adjusted accordingly. The authors state that the scientific conclusions are unaffected. This correction was approved by the Academic Editor. The original publication has also been updated.

The newly added reference appears below:

36. Anders, M.; Dreismickenbecker, E.; Fleckenstein, J.; Walter, C.; Enax-Krumova, E.K.; Fischer, M.J.; Zinn, S. EEG-based sensory testing reveals altered nociceptive processing in elite endurance athletes. *Exp. Brain Res*. **2022**, *241*, 341–354. https://doi.org/10.1007/s00221-022-06522-4.
